# Steroids Up-Regulate p66Shc Longevity Protein in Growth Regulation by Inhibiting Its Ubiquitination

**DOI:** 10.1371/journal.pone.0015942

**Published:** 2011-01-14

**Authors:** Santosh Kumar, Satyendra Kumar, Mythilypriya Rajendran, Syed Mahfuzul Alam, Fen-Fen Lin, Pi-Wan Cheng, Ming-Fong Lin

**Affiliations:** 1 Department of Biochemistry and Molecular Biology, College of Medicine, University of Nebraska Medical Center, Omaha, Nebraska, United States of America; 2 Eppley Institute for Cancer Research, University of Nebraska Medical Center, Omaha, Nebraska, United States of America; Florida International University, United States of America

## Abstract

**Background:**

p66Shc, an isoform of Shc adaptor proteins, mediates diverse signals, including cellular stress and mouse longevity. p66Shc protein level is elevated in several carcinomas and steroid-treated human cancer cells. Several lines of evidence indicate that p66Shc plays a critical role in steroid-related carcinogenesis, and steroids play a role in its elevated levels in those cells without known mechanism.

**Methods and Findings:**

In this study, we investigated the molecular mechanism by which steroid hormones up-regulate p66Shc protein level. In steroid-treated human prostate and ovarian cancer cells, p66Shc protein levels were elevated, correlating with increased cell proliferation. These steroid effects on p66Shc protein and cell growth were competed out by the respective antagonist. Further, actinomycin D and cyclohexamide could only partially block the elevated p66Shc protein level by steroids. Treatment with proteasomal inhibitors, but not lysosomal protease inhibitor, resulted in elevated p66Shc protein levels, even higher than that by steroids. Using prostate cancer cells as a model, immunoprecipitation revealed that androgens and proteasomal inhibitors reduce the ubiquitinated p66Shc proteins.

**Conclusions:**

The data collectively indicate that functional steroid receptors are required in steroid up-regulation of p66Shc protein levels in prostate and ovarian cancer cells, correlating with cell proliferation. In these steroid-treated cells, elevated p66Shc protein level is apparently in part due to inhibiting its ubiquitination. The results may lead to an impact on advanced cancer therapy via the regulation of p66Shc protein by up-regulating its ubiquitination pathway.

## Introduction

Shc (Src homolog and collagen homolog) proteins are identified as adaptor molecules mediating tyrosine phosphorylation signaling [1]. ShcA, the Shc proteins in mammalian cells, exists in three different isoforms with molecular masses of 46, 52 and 66 kDa. All isoforms contain three functional domains – an SH2 domain, a PTB domain and a CH1 domain with three conserved tyrosine residues that are phosphorylated in response to various signals [1]. Additionally, p66Shc has a unique CH2 domain at the N-terminus, which contains a serine residue (Ser-36) that can be phosphorylated under stress signals [2]. Different members of the Shc proteins exhibit distinct expression patterns and biological functions. For example, p52Shc and p46Shc are expressed in most cells, while p66Shc protein is expressed predominantly in epithelial cells [3]. Both p52Shc and the majority of p66Shc are distributed throughout the cytosol, whereas a fraction of p66Shc and p46Shc localize to mitochondria [4], [5]. Shc proteins were first described as adaptor proteins that bridge the growth factor receptor-bound protein (grb2)-son of seven less (sos1) complex to the phosphorylated receptor tyrosine kinase (RTK), resulting in activation of the membrane-bound GTPase ras [6]. Thus, Shc protein plays critical roles in diverse signal pathways.

p66Shc is unique among ShcA proteins because of its distinct structural and functional features [5]. Functionally, p66Shc, but not other two ShcA proteins, play a pivotal role in regulating the intracellular level of reactive oxygen species (ROS) [5], [7]. By virtue of its ability to modulate ROS levels, p66Shc plays an important role in the aging and age-associated bioprocesses including, for example, vascular dysfunction [8]. In mammals, p66Shc functions as a longevity gene [2]. Nevertheless, its role in human longevity requires further investigation.

Despite the fact that results of many studies indicate p66Shc as a mediator of apoptosis, recent advances associate p66Shc with human epithelial cell proliferation and carcinogenesis [5]. For example, in ovarian carcinoma cell lines, p66Shc protein level positively correlates with ErbB-2 expression, a prognostic marker for ovarian cancer [9]. In breast cancer, p66Shc protein level is increased in cell lines with highly metastatic ability and is elevated in lymph node-positive tumors [10]. Nevertheless, a negative correlation between p66Shc expression and primary tumor of breast cancer has been reported [11], [12]. It should be noted, in that study many specimens from patients under hormone therapy were utilized [12]. Further studies are thus required to define its role in breast carcinogenesis. Importantly, in prostate, ovarian, thyroid and colon carcinoma tissues, p66Shc protein levels are higher in cancerous cells than that in the adjacent non-cancerous cells [10], [13], [14], [15], [16]. In prostate cancer cell lines, p66Shc protein level positively correlates with their growth rates [14], [17]. Further, growth stimulation of prostate, testis and breast cancer cell lines with respective steroid hormones is accompanied by an increase of p66Shc protein level [14], implying its function in steroid-induced proliferation. Evidently, p66Shc knockdown is associated with diminished cell growth [17]. Thus, p66Shc signaling plays a functional role in regulating the proliferation and the carcinogenesis of diverse cell types. However, the regulatory mechanism of p66Shc protein level related to its function remains an enigma.

The expression level of a protein can be controlled through the regulation of its transcription, translation and stability via degradation process. Although several factors including epigenetic regulation have been reported to affect p66Shc expression [18], [19], [20], the specific mechanisms underlying how p66Shc protein, the functional molecule, is regulated remain to be elucidated. Targeting of cellular proteins for proteasomal proteolysis marked by ubiquitination is a highly complicate and tightly regulated process [21]. Protein ubiquitination is a signal for target recognition and ATP-dependent proteolysis by the 26S proteasome [22], [23], [24]. Ubiquitin, an evolutionarily conserved protein of 76 residues, exhibits diverse cellular functions. It can be covalently conjugated to lysine residues in target proteins including marking mono or polyubiquitination of proteins [24]. Polyubiquitination of proteins in general is widely acknowledged as a signal for degradation by the proteasome [25], while monoubiquitination, although presently ill-defined, has been shown to influence numerous cellular events, including endocytosis and meiosis [26], [27]. Since in steroid hormones-treated cells, p66Shc protein levels are elevated, correlating with cell proliferation, we investigate the molecular mechanism by which steroids up-regulate p66Shc protein. Understanding this machinery may lead to develop therapeutic strategies toward steroid-regulated cancers.

## Materials and Methods

### Reagents and antibodies

Fetal bovine serum (FBS), gentamicin, L-glutamine and RPMI 1640 medium were obtained from Invitrogen (Carlsbad, CA, USA). Charcoal/dextran-treated FBS was purchased from Atlanta Biologicals (Lawrenceville, GA, USA). *N*-carbobenzoxyl-L-leucinyl-L-leucinyl-L-norleucinal (MG 132), lactacystin, leupeptin and actinomycin D (Act D) were from Calbiochem (San Diego, CA). Polyclonal antibody (Ab) recognizing all three Shc isoforms of Shc protein was from Upstate Biotechnology Inc. (Lake Placid, NY, USA). Horseradish peroxidase-conjugated anti-mouse and anti-rabbit IgG Abs were from Santa Cruz Biotechnology (Santa Cruz, CA). Anti-ubiquitin and anti-β-actin Ab, 5α-dihydrotestoesteron (DHT), estrogen (E2), tamoxifen, cyclohexamide (CHX), protease inhibitor cocktail, pancreatic bovine insulin, Protein A-Sepharose coated beads were purchased from Sigma (St Louis, MO, USA). All other chemicals were as described previously [14], [17], [28], [29].

### Cell lines

LNCaP-FGC human prostate cancer cells, CaOV-3 and OVCAR-3 human ovarian cancer cell lines were originally purchased from the American Type Culture Collection (Rockville, MD, USA). LNCaP C-33 cells are androgen-sensitive and routinely maintained in phenol red-positive RPMI 1640 medium supplemented with 5% FBS (v/v), 2 mM glutamine and 50 µg/ml gentamicin [28]. CaOV-3 cells are routinely maintained in phenol red-positive RPMI 1640 medium supplemented with 10% FBS (v/v), 2 mM glutamine and 50 µg/ml gentamicin. OVCAR-3 cells are routinely maintained in phenol red-positive RPMI 1640 medium supplemented with 20% FBS (v/v), 2 mM glutamine, pancreatic bovine insulin (10 µg/ml) and gentamicin (50 µg/ml). Cells were split once per week, which was defined as one passage. LNCaP cells with passage numbers less than 33 were designated as C-33 [28], [30]. For DHT and E2 treatments, LNCaP C-33, CaOV-3 and OVCAR-3 cells were steroid-starved for 48 h in a steroid-reduced medium, i.e., phenol red-free RPMI 1640 medium containing 5% charcoal/dextran-treated FBS (v/v), 2 mM glutamine and gentamicin (50 µg/ml). LNCaP cells were exposed to DHT (10 nM), CaOV-3 and OVCAR-3 cells were exposed to E2 (10 nM), and cells were harvested after various periods of time, as indicated in each experiment. For treating with RNA and protein *de novo* biosynthesis inhibitors, cells were pretreated for 1 h with Act D and CHX prior to the addition of DHT in the same medium for an additional 24 h.

### Immunoblotting

Briefly, subconfluent cells were gently rinsing twice with ice-cold 20 mM HEPES-buffered saline, pH 7.0, scraped, and cell pellet was lysed in ice-cold high-stringent cell lysis buffer containing protease and phosphatase inhibitors. The detailed protocols for immunoblotting were described previously [14], [28], [29]. For semi-quantifying the intensity of hybridization bands, the ImageJ.exe program (http://rsb.info.nih.gov) was used.

### Cell growth determination

To determine the growth rate, cells were seeded on six-well culture plates and maintained in their respective culture medium. At the specific time point, attached cells was harvested by trypsinization and the cell number was measured by a cell counter cellometer™ Auto T4 (Nexcelom Bioscience, USA).

### Immunoprecipitation

To immunoprecipitate p66Shc protein, cells were washed with ice-cold 20 mM HEPES-buffered saline, pH 7.0, collected and pelleted by centrifugation, and lysed on ice for 20 min with lysis buffer (50 mM Tris, pH 7.4, 150 mM NaCl, 5 mM EDTA, 0.5% NP-40, protease inhibitor cocktail). Abs to Shc protein (3 µg) and to ubiquitin (20 µl) were first respectively incubating with Protein A-Sepharose beads (50 µl of 10% suspension) in 500 µl of lysis buffer for 1 h at 4°C. Cell lysates (0.3 mg) were incubated with Abs coated in Protein A-Sepharose beads in a volume of 500 µl at 4°C for 2 h. Beads were washed three times (1 ml each) with ice-cold lysis buffer. Immunoprecipitated proteins were eluted by heating at 95°C for 5 min in Laemmli sample buffer (50 mM Tris HCl, pH 6.8, 2% SDS (v/v), 0.001% bromophenol blue, 10% glycerol (v/v), 100 mM dithioerithreitol) [31].

### Statistical analysis

Each set of experiments was repeated at least 3 times and the mean and standard error values were calculated. The significance of difference (P-value) was calculated using independent t-test and the P-value less than 0.05 was considered as significant.

## Results

### Effects of steroids on p66Shc protein levels and the proliferation of prostate and ovarian cancer cells

**Figure 1:**
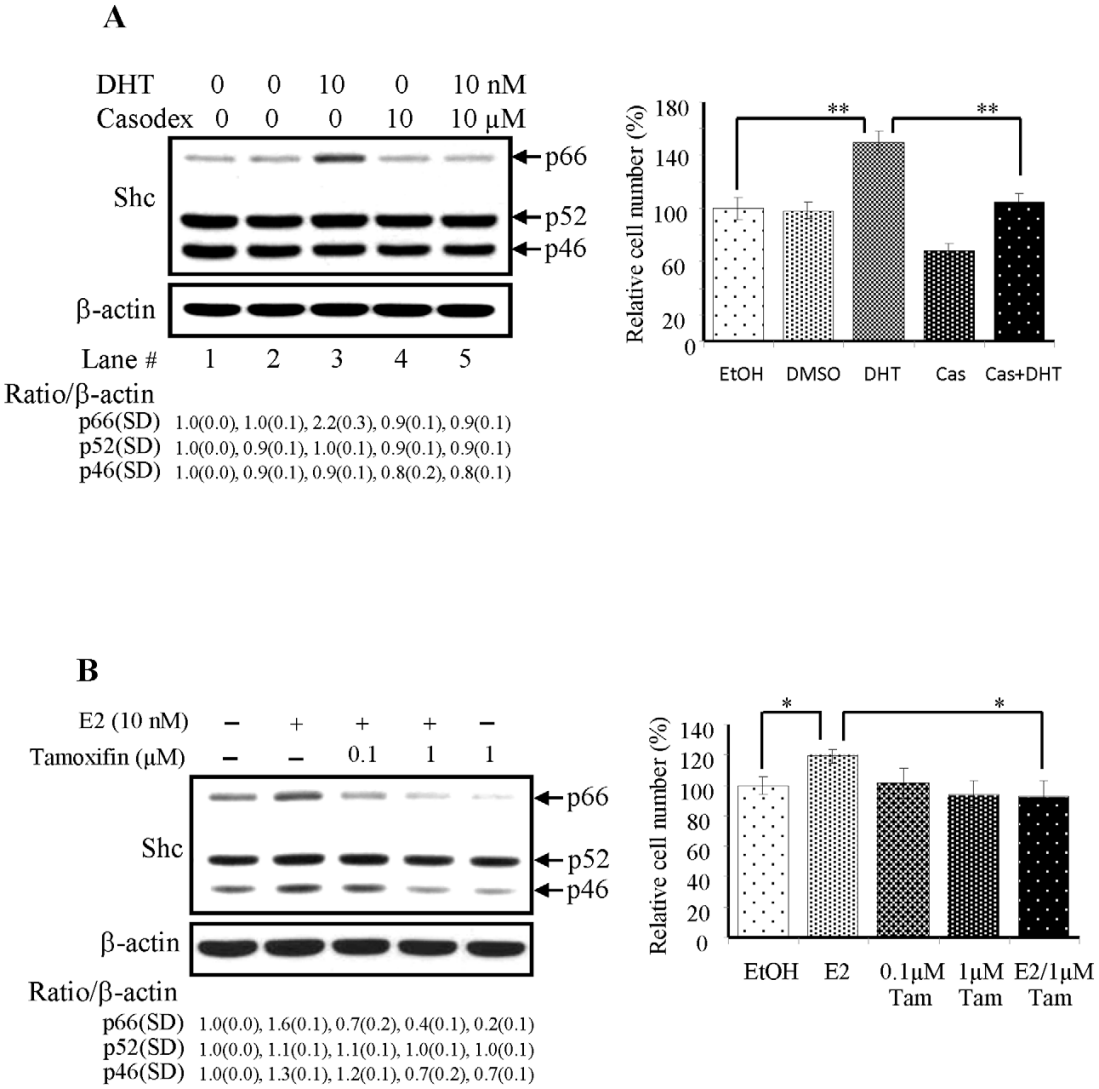
Effects of steroids on p66Shc protein levels in PCa and OVa cells and their proliferation. LNCaP C-33 and CAOV-3 cells were plated in duplicates in the regular medium as described in the methods for 72 h and then maintained in a steroid-reduced medium for 48 h. Cells were treated with steroids plus or minus the corresponding antagonists as specified in each figure for 48 h. After harvested, lysates were analyzed by western blotting with Abs against total Shc and β-actin protein, respectively. The level of β-actin protein was detected as a loading control. The intensity of each Shc protein hybridization band was semiquantified, and the ratio to the corresponding β-actin protein was calculated and then normalized to that of corresponding control cells which received the solvent alone. To determine cell growth, cell number was analyzed by cell counting. The figure is a representative of three sets of independent experiments in duplicates and the cell growth is expressed as means ± SE (*n* = 3). (**A**) LNCaP C-33 cells were treated with or without DHT (10 nM), plus or minus casodex (10 µM) for 48 h. Left panel: Lane 1, cells received EtOH as a control; Lane 2, cells received DMSO as a control; Lane 3, cells received DHT in EtOH; Lane 4, cells received casodex in DMSO; Lane 5, cells received both DHT & casodex. Right panel: Cell growth was analyzed by cell counting. (n = 2×3, **P<0.001). (**B**) Left panel: CaOV-3 cells treated without or with E2 (10 nM), tamoxifen (0.1 and 1 µM) for 48 h. Right panel: Cell growth was analyzed by cell counting. (n = 2×3, *P<0.05). SD, Standard deviation.

LNCaP C-33 cells are androgen-sensitive cells with a slow growth rate and express a low level of p66Shc protein. Since p66Shc protein level can be up-regulated by steroid hormones [14], we investigated the molecular mechanism by which p66Shc protein is elevated in DHT-treated LNCaP cells. As shown in Fig. 1A (lane #3 of left panel), in DHT-treated LNCaP C-33 cells, p66Shc protein levels, but not p52 or p46 Shc protein, were elevated as observed previously [14]. The hybridization band was semiquantified by scanning and then normalizing to that of β-actin. The data validated the specific DHT effect on p66Shc protein, but not p52 or p46Shc, by over 2-fold elevation (lane #3, Fig. 1A). Concurrently, the proliferation of DHT-treated cells was increased (Fig. 1A, column #3 of right panel). On contrast, in the presence of casodex, an AR antagonist used in clinical androgen ablation therapy, the elevation of p66Shc protein by DHT was abolished (Fig. 1A, lane #5 of left panel) and cell growth was diminished (Fig. 1A, column #5 of right panel). Thus, casodex blocks the up-regulatory effect of DHT on p66Shc protein as well as the growth of LNCaP C-33 cells. The data indicate that AR activity is required for androgen-mediated up-regulation of p66Shc protein level.

We further investigated whether E2 also could up-regulate p66Shc protein level in ovarian cancer cells as that by DHT in prostate cancer cells. Ovarian cancer CaOV-3 cells are estrogen-sensitive cells and express a high basal level of p66Shc protein. In E2-treated CaOV-3 cells, p66Shc protein levels were elevated, higher than that in control cells (Fig. 1B, left panel). The only approximately 50% elevation by E2 was at least in part due to the high basal level in the absence of E2 (Fig. 1B). In the presence of tamoxifen, an antagonist of estrogen receptor (ER), the elevated p66Shc level was reduced in a dose-dependent fashion (Fig. 1B, left panel). The specific effect by E2 on p66Shc protein level was further validated by semiquantifying the hybridization bands (Fig. 1B, left panel). In parallel, E2 promoted cell growth, which was competed out by tamoxifen (Fig. 1B, right panel). Thus, tamoxifen abolishes the up-regulatory effect of E2 on p66Shc protein in CaOV-3 cells, indicating that ER activity is required for E2-elevated p66Shc protein levels as well as cell proliferation. The data together show that steroids via the corresponding receptors up-regulate p66Shc protein levels, correlating with stimulated cell proliferation.

### Effects of *de novo* biosynthesis inhibitors on p66Shc protein level by androgens

**Figure 2:**
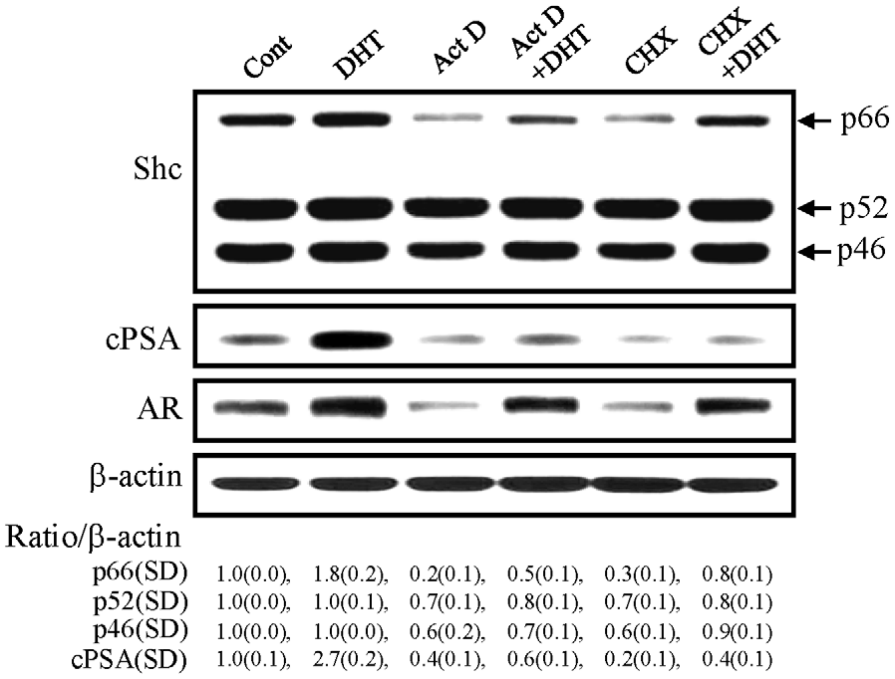
Effects of *de novo* biosynthesis inhibitors on p66Shc protein levels. LNCaP C-33 cells were plated in the regular culture medium for 72 h. After steroid starvation for 48 h, cells were treated with or without DHT (10 nM), 5 µg/ml actinomycin D (Act D) or 10 µg/ml cyclohexamide (CHX) as specified in the figure for 24 h. After harvesting, cell lysates were analyzed by western blotting with Abs against total Shc, prostate-specific antigen (PSA), androgen receptor (AR) and β-actin protein, respectively. The level of β-actin protein was detected as a loading control. The intensity of p66Shc hybridization band was semiquantified, and the ratio to the corresponding β-actin protein was calculated and then normalized to that of control LNCaP cells which received the solvent alone. The figure is a representative of four sets of independent experiments. SD, Standard deviation.

p66Shc protein plays a critical role in steroid-stimulated cell growth [32]. Due to the importance of p66Shc protein in growth regulation, we investigated whether androgens up-regulated the biosynthesis of p66Shc protein. In LNCaP C-33 cells, in the absence of androgen, 80% and 70% of the basal p66Shc protein level was reduced by Act D and CHX, inhibitors of *de novo* RNA synthesis and *de novo* protein synthesis, respectively (Fig. 2). In this set of experiments, in the presence of DHT alone, p66Shc protein level was elevated by about 2 folds, and Act D and CHX could only have a partial effect on reducing DHT-induced p66Shc protein levels in these cells. In comparison, DHT treatment increased cPSA, an androgen-regulated protein, by up to 3-fold and Act D and CHX treatments resulted in a great decrease of PSA (Fig. 2). Interestingly, in the presence of DHT, Act D and CHX had only marginal effects on decreasing AR protein levels. Thus the data indicated that in DHT-treated cells, the elevation of p66Shc protein level could not be explained by *de novo* biosynthesis alone.

### Effects of proteasomal and lysosomal protease inhibitors on p66Shc protein level in PCa cells

**Figure 3:**
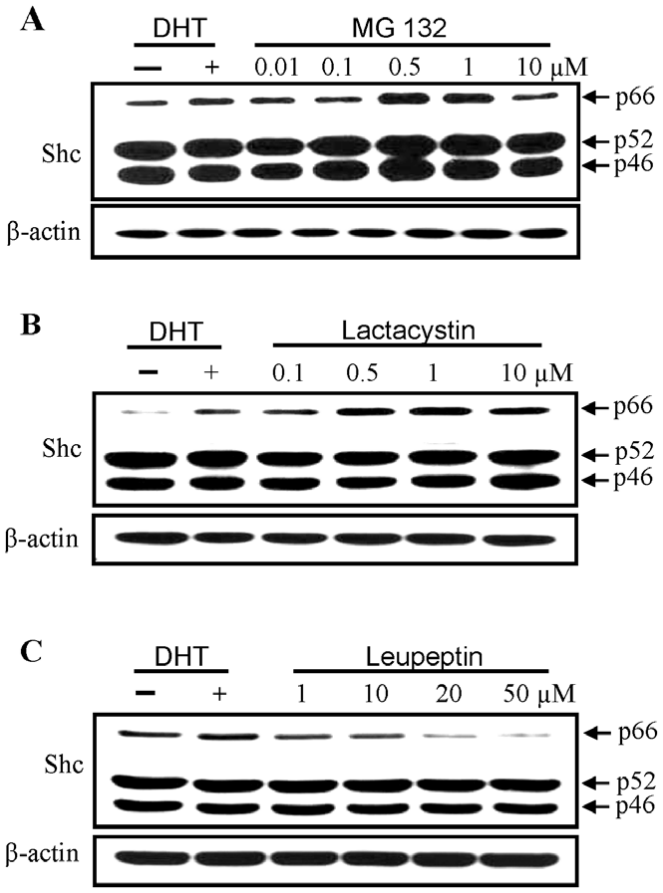
Dosage effects of proteasomal inhibitors on p66Shc protein levels. LNCaP C-33 cells were seeded in regular culture medium for 72 h, steroid starved for 48 h and then cells were treated with or without 10 nM DHT or different concentrations of (**A**) MG132 (0.01 to 10 µM), (**B**) lactacystin (0.1 to 10 µM) and (**C**) leupeptin (1 to 50 µM) for 24 h. Cells were harvested and lysates were analyzed by western blotting with Abs against total Shc protein and β-actin protein, respectively. The level of β-actin protein was detected as a loading control. The figure is a representative of at least five sets of independent experiments.

We investigated the involvement of protein degradation pathway in up-regulating p66Shc protein level by DHT. p66Shc protein was analyzed in the presence of proteasomal inhibitors MG 132 (0.01 to 10 µM) and lactacystin (0.1 to 10 µM) and a lysosomal protease inhibitor leupeptin (1 to 50 µM) under a steroid-reduced condition. As a positive control, cells were treated with 10 nM DHT. As shown in Fig. 3A, p66Shc protein level was elevated in the presence of proteasomal inhibitors, essentially following a bell-shaped dose-dependent fashion. In MG 132- and lactacystin-treated cells, the p66Shc protein level was even higher than that in DHT-treated cells. Nevertheless, leupeptin treatment didn't cause an elevation of p66Shc protein level (Fig. 3B). Instead, the p66Shc protein level was decreased at high concentrations of leupeptin; while leupeptin did not have an effect on p52Shc, p46Shc or β-actin protein levels. The data indicate that DHT increases p66Shc protein level may in part via the inhibition of proteasomal pathway.

### Kinetic analyses of androgens and proteasomal inhibitors on p66Shc protein level in PCa cells

**Figure 4:**
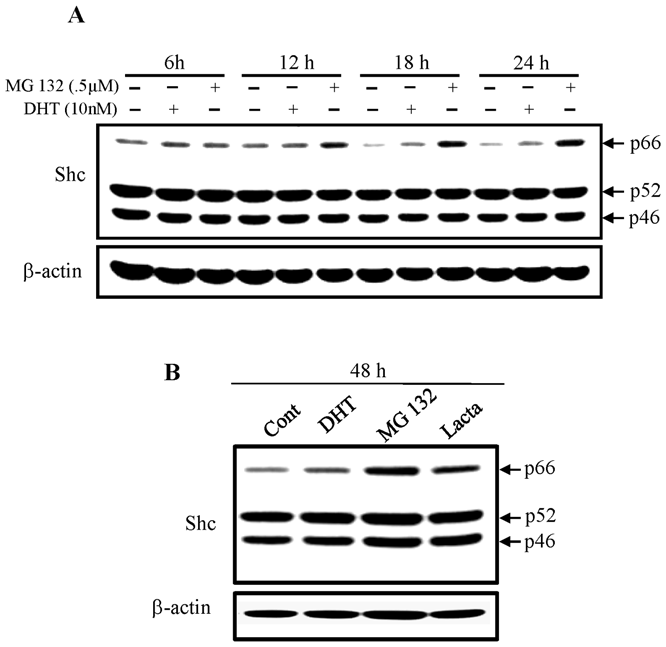
Kinetic analyses of DHT and proteasomal inhibitors on p66Shc protein levels. LNCaP C-33 cells were plated in regular medium for 72 h, steroid starved for 48 h, and then cells were treated without or with DHT (10 nM), MG 132 (0.5 µM) or Lactacystin (1 µM) for various time periods as indicated in the figure. Cells were harvested and lysates were analyzed by western blotting with Abs against total Shc protein and β-actin protein, respectively. The level of β-actin protein was detected as a loading control. The figure is a representative of three sets of independent experiments. (**A**) Cells were treated with DHT (10 nM) or MG132 (0.5 µM) and harvested at 6 h, 12 h, 18 h and 24 h and (**B**) cells were treated with DHT (10 nM), MG132 (0.5 µM) or Lactacystin (1 µM)) and harvested at 48 h after treatment.

Effects of DHT and proteasomal inhibitors on p66Shc protein levels were examined kinetically in LNCaP C-33 cells. As shown in Figs. 4A and 4B, p66Shc protein levels were elevated in DHT-, MG132- and lactacystin-treated cells, following the time course. Semiquantitative analyses of the intensity of p66Shc protein hybridization bands revealed that the level of p66Shc protein was elevated starting at 6 h after treatment by both DHT and MG 132 and through 48 h treatment. Further, p52Shc and p46Shc protein levels were remained essentially the same. Thus, the observed elevation of p66Shc by androgens in LNCaP cells is at least in part regulated at the posttranslational level.

### Effect of estrogens and proteasomal inhibitors on p66Shc protein level in ovarian carcinoma cells

**Figure 5:**
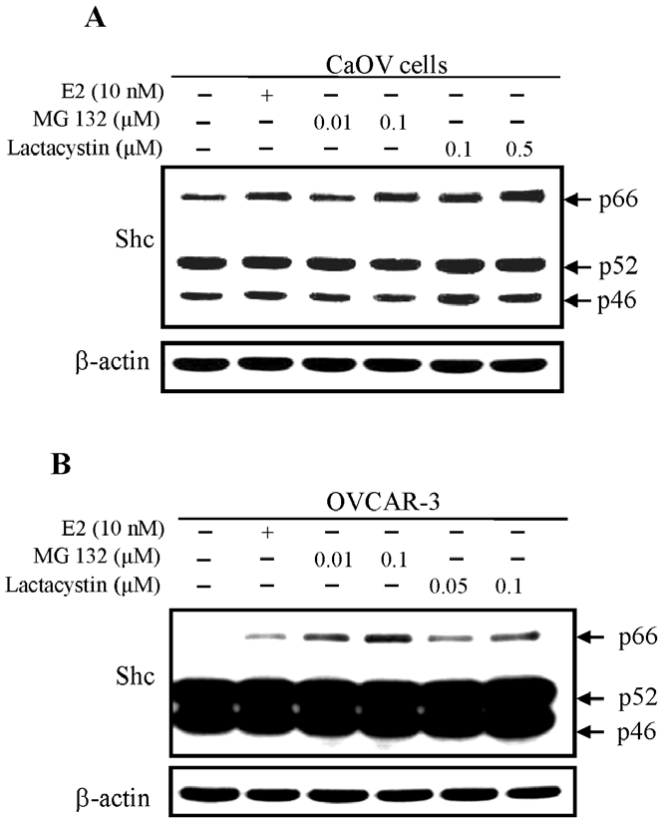
Effects of E2 and proteasomal inhibitors on p66Shc protein levels in ovarian carcinoma cells. CaOV-3 and OVCAR-3 cells were plated in regular medium for 72 h and steroid starved for 48 h. (**A**) CaOV-3 cells were treated with or without E2 (10 nM), MG 132 (0.01, 0.1 µM) or Lactacystin (0.1, 0.5 µM) for 48 h and (**B**) OVCAR-3 cells were treated with or without E2 (10 nM), MG 132 (0.01, 0.1 µM) or Lactacystin (0.05, 0.1 µM) for 48 h. Cells were harvested and lysates were analyzed by western blotting with Abs against total Shc protein and β-actin protein, respectively. The level of β-actin protein was detected as a loading control. The figure is a representative of three sets of independent experiments.

We investigated whether proteasomal inhibitors also could have an effect on p66Shc protein level in ovarian cancer cells. As shown in Fig. 5A, despite a high basal level in the absence of E2, p66Shc protein levels were further elevated in E2- and proteasomal inhibitors-treated CaOV-3 cells. In OVCAR-3 cells, another estrogen-sensitive ovarian cancer cell line that expresses a very low level of p66Shc, E2 treatment greatly increased p66Shc protein level. Similarly, in the presence of proteasomal protease inhibitors, p66Shc protein level elevated, even higher than that by E2 (Fig. 5). Thus, in E2- and proteasomal inhibitors-treated ovarian cancer cells, p66Shc protein levels were elevated, apparently due to reduced degradation.

### Androgen effect on the ubiquitination of p66Shc protein

**Figure 6:**
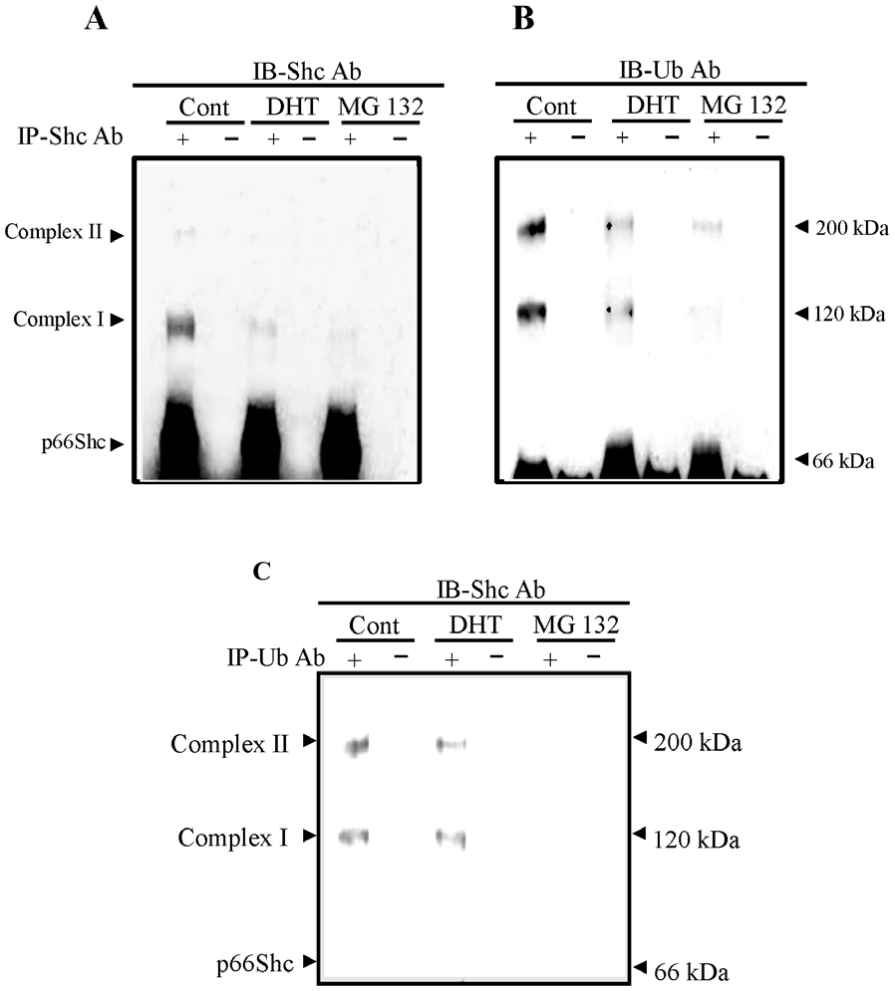
Androgen effects on the ubiquitination of p66Shc proteins. LNCaP C-33 cells were plated in regular medium for 72 h, steroid starved for 48 h and then treated with or without DHT (10 nM) and MG 132 (0.5 µM) as a positive control for 24 h. Cell were harvested in lysis buffer. Total cellular lysates (300 µg proteins) were reacted with (**A & B**) anti-Shc or (**C**) anti-Ubiqutin Ab (3 µg each), and followed by Protein A–Sepharose beads. The immune complexes were analyzed by immunoblotting with (**A**) anti-Shc Ab, (**B**) anti-ubiquitin Ab and (**C**) anti-Shc Ab. The positions of p66Shc and p66Shc/Ub-protein complex are indicated by arrow-heads. The figure is a representative of four sets of independent experiments.

Since DHT and E2 treatments up-regulate p66Shc protein level at least in part via the same pathway, we used LNCaP C-33 cells as the model system for further analysis. We investigated DHT effects on the proteasomal degradation pathway in LNCaP C-33 cells by analyzing ubiquitinated-p66Shc protein complexes. MG 132-treated LNCaP C-33 cells were used as a control. p66Shc protein in DHT- and MG 132-treated LNCaP C-33 cell lysates were immunoprecipitated by Abs to Shc proteins and the immunoprecipitated complex was analyzed by immunoblotting with anti-Shc Abs and anti-ubiquitin Abs, respectively. Under the steroid-reduced condition, p66Shc protein was highly ubiquitinated as indicated by the appearance of high mol wt. complexes that were reacted with anti-ubiquitin Ab (Fig. 6B, control). The ubiquitinated-p66Shc protein complexes were greatly decreased in DHT- and MG 132-treated LNCaP cells (Fig. 6A & 6B). Semiquantitative analyses revealed that over 70% of complex I were decreased in DHT- and MG132-treated cells, respectively (Figs. 6A and 6B). To further confirm the ubiquitination of p66Shc protein in the SR-condition, total ubiquitinated proteins were immunoprecipitated by an anti-ubiquitin Ab followed by immunoblotting with Abs specific to Shc proteins. Western blot analyses revealed the presence of high molecular weight p66Shc in immunoprecipitated ubiquitin-conjugated proteins, which was reduced by DHT, although to a lesser degree than that shown in Figs. 6A and 6B, and was abolished by MG 132 (Fig. 6C). These data together indicate that in DHT-treated LNCaP C-33 cells, the ubiquitinated p66Shc protein is decreased, as seen in the proteasome inhibitors-treated cells.

## Discussion

p66Shc protein plays a critical role in regulating oxidative stress response and life span in mammals. It is also shown to be correlated with the progression of several steroid-regulated tumors [5]. Due to the potential importance of p66Shc protein involved in diverse signal activities including steroid-regulated carcinogenesis, we investigate the molecular mechanism by which steroid hormones up-regulate p66Shc protein level.

Our data clearly show that DHT treatment of androgen-sensitive prostate cancer LNCaP C-33 cells leads to an increase in p66Shc protein, but not p52Shc or p46Shc protein, and cell proliferation (Fig. 1A) [14]. This up-regulatory effect of DHT on p66Shc and cell growth is abolished by casodex (Fig. 1A), an antagonist to AR in clinical androgen-ablation therapy for advanced PCa [33]. Apparently, AR activity is required in DHT up-regulating p66Shc protein. Similarly, E2 up-regulation of p66Shc protein levels in ovarian cancer cells (Fig. 1B) and breast carcinoma cells [14] require functional ER, correlating with cell proliferation (Fig. 1B) [14]. The data collectively indicate that p66Shc plays a critical role in regulating the growth rate of these cells [17], [32]. Thus, it is imperative to delineate steroid regulation of its protein. Interestingly, while Act D can greatly block DHT effect on elevating p66Shc and cPSA protein levels; CHX can only have a partial inhibitory activity on DHT-induced p66Shc protein, differing from CHX effect on DHT-induced cPSA protein level (Fig. 2). Since DHT and E2 up-regulating p66Shc proteins are not entirely through *de novo* biosyntheses alone (Fig. 2), androgens and estrogens up-regulate p66Shc protein at least in part via post-translational regulation.

Previous studies indicated that tumor suppresser p53 protein increases p66Shc protein stability in mouse embryonic fibroblast cells, apparently signaling for apoptotic pathway [34]. Nevertheless, our study revealed that DHT treatment does not increase p53 protein levels in LNCaP C-33 cells (data not shown) [32], whereas DHT increases p66Shc protein levels and cell proliferation. p53 is a tumor suppressor and its expression is either down regulated or the protein is mutated in about 60% of advanced cancer cells [35], whereas p66Shc protein level is elevated in several types of cancers [5], [14], [15], [16]. In this study, DHT and E2 respectively up-regulate p66Shc protein level in PCa and OVa cells. In these growth-stimulated cells, p53 protein would not be elevated, while p66She is elevated. These data together indicate that DHT and E2 increase p66Shc protein level in LNCaP and ovarian cancer cells, respectively, via a p53-independent manner.

Ser36 phosphorylation of p66Shc is proposed to signal the translocation of p66Shc to mitochondria for apoptotic pathway [36]. In the steroid-reduced condition, as an androgen-sensitive cell, Ser36 phosphorylation at p66Shc protein is at a high level in LNCaP C-33 cells (data not shown) [32]. This elevated phosphorylation is decreased in the presence of DHT, a survival factor for those cells, and p66Shc protein level is elevated. Interestingly, in the presence of proteasomal inhibitors, Ser36 phosphorylation is also diminished without known mechanism (data not shown). Alternatively, Ser54 and Ser286 phosphorylation may play a role in steroid-induced p66Shc protein stability [20]. Further investigation is thus required to determine the functional amino acid residue in regulating its protein stability in different cell types under different growth conditions.

Interestingly, steroids can interact with the proteasomal degradation pathway, leading to the activation of AR signaling [37]. Our results (Figs. 3 and 4) clearly show that in the presence of proteasome inhibitors MG 132 and lactacystin, but not lysosomal protease inhibitor leupeptin, p66Shc protein level is elevated to a level that is even higher than DHT effects. In the presence of proteasomal inhibitors or steroids, p66Shc protein levels are elevated in prostate and ovarian cancer cells, following a dose-dependent and a time course-dependent fashion. These data clearly indicate that p66Shc is degraded by the proteasomal pathway, and steroid hormones may prevent p66Shc protein from degradation.

The ubiquitin-proteasome pathway is a major pathway for intracellular protein degradation. Protein substrates are first “marked” with poly-ubiquitin chains and then degraded to peptides and free ubiquitin by a large multimeric protease, the proteasome, which exists within all eukaryotic cells [38]. There are two known sequences that are required for proteasomal degradation. One is the PEST sequence [39] and the other is the destruction box [40]. While there is no identifiable destruction box within the sequence of p66Shc protein, p66Shc has two PEST motifs encompassing amino acids 14 through 64 in the CH2 domain and amino acids 328 through 347 in the CH1 domain [20]. Interestingly, Ser36 and Ser54 are located within the first PEST motif. Further experiments are required to clarify if both PEST motifs are responsible for p66Shc degradation.

Our results reveal that ubiquitinated p66Shc proteins in LNCaP C-33 cells are of high molecular mass (>100 kDa) (Fig. 6). Such large conjugates have been shown in vitro to be preferentially degraded by the 26S (1500 kDa) proteasome complex [41], [42], [43], [44]. Furthermore, ubiquitinated p66Shc proteins are decreased in DHT- and MG132-treated cells (Fig. 6), whereas the amounts of the non-ubiquitinated 66 kDa proteins increase in these cells (Figs. 3 & 4). The data also indicate that such high molecular mass of Ub-protein conjugates accumulate rapidly (observed at 6 h) in steroid-reduced, stressful condition in which most cellular activities are suppressed, comparing to its half life of 4.5 h in regular growth condition [20]. These high-molecular-mass conjugates apparently represent the multi poly-ubiquitinated p66Shc proteins marked for degradation. Further experiments should determine the molecular structure of these complexes. These data together support the notion that DHT-induced elevation of p66Shc protein level in LNCaP C-33 cells is through inactivation of proteasomal pathway by inhibiting its ubiquitination. Similarly, E2 modulates p66Shc protein levels in ovarian cancer cells apparently by preventing it from proteasomal degradation (Fig. 5). These observations also raise an interesting question if steroids and proteasomal inhibitors can also suppress the ubiquitination process for blocking the proteasomal activity. Our data thus reveal a novel non-genomic regulatory mechanism by steroid hormone on p66Shc protein, a functional protein involving in diverse cellular activities. Due to the potential importance of p66Shc in carcinogenesis and tumor progression of diverse cancer types, targeting to the proteasomal pathway to facilitate p66Shc degradation can be an alternative approach to advanced cancer therapy.
